# Data Hidden in Sewage: Advanced Methods for Identification and Quantification of Synthetic Cannabinoids in Urban Wastewater

**DOI:** 10.3390/molecules31020337

**Published:** 2026-01-19

**Authors:** Wiktoria Kurzeja, Mariola Kuczer, Jan Matysiak, Agnieszka Klupczyńska-Gabryszak

**Affiliations:** 1 Faculty of Chemistry, University of Wrocław, F. Joliot-Curie 14, 50-383 Wrocław, Poland; wiktoriakurzeja23@gmail.com (W.K.); mariola.kuczer@uwr.edu.pl (M.K.); 2 Department of Inorganic and Analytical Chemistry, Faculty of Pharmacy, Poznan University of Medical Sciences, Rokietnicka 3, 60-780 Poznań, Poland; jmatysiak@ump.edu.pl

**Keywords:** wastewater, sewage, wastewater-based epidemiology, sewage-based epidemiology, synthetic cannabinoids

## Abstract

Synthetic cannabinoids (SCs) represent one of the rapidly growing groups of new psychoactive substances (NPS) on the illicit drug market. SCs mimic the effects of Δ^9^-tetrahydrocannabinol, but they have a greater affinity to the receptors, resulting in more potent psychoactive effects than traditional substances. The toxicity and high abuse potential of SCs could pose serious health risks to their users. The challenges posed by the SCs require innovative monitoring strategies like the analysis of untreated wastewater, known as wastewater-based epidemiology (WBE). In this review article, we summarized the available literature on the detection and quantification of SCs in raw wastewater samples published between 2013 and 2025. We paid special attention to challenges related to different experimental stages of WBE analysis that hinder the accurate measurement of SCs and their metabolites. The reviewed studies show that wastewater analysis reflected the dynamic evolution of the illicit SCs market. As studies on the analysis of SCs in wastewater remain scarce, large monitoring campaigns and research performed in more locations are needed. Modern analytical hyphenated systems such as LC-MS are essential for the sensitive and accurate quantification of SC biomarkers in wastewater and their sound identification. Future studies should address further stability tests, investigation of SC metabolism, and careful selection of the effective SC extraction method from the complex environmental matrix.

## 1. Introduction

Synthetic cannabinoids (SCs) represent one of the rapidly growing groups of new psychoactive substances (NPSs) on the illicit drug market. Since 2024, the European Union Drug Agency (EUDA) has been monitoring 247 SCs, which makes them the largest group of NPS in Europe [1]. They are difficult to detect and monitor because of their chemical diversity and the speed of their emergence.

Products of SCs are marketed as herbal smoking mixtures in attractive packages with appealing names such as “K2” (in North America), “Spice” (in Europe), or “Youcatan”, “Chill”, or “Black Mamba”. SCs may also be available in alternative formulations, including powders, tablets, and e-liquids for vaping. They are sold mostly over the Internet or on the street market [2,3,4].

Most of these products are distributed to the European market from China [3]. During the production, SCs are dissolved in an organic solvent and subsequently applied to plant material through spraying or directly mixed with it. These herbal mixtures may contain unidentified compounds or other psychoactive substances (e.g., ecstasy) as well as toxicants such as pesticides or rodenticides, which may contribute to their adverse effects. The exact composition and toxicity of these products vary widely. They typically contain more than one SC and the composition is varied to avoid legal constraints. For example, analyses showed that the concentration of JWH-018 in a portion ranges from 0.2 mg/g to 47 mg/g [5].

**Figure 1:**
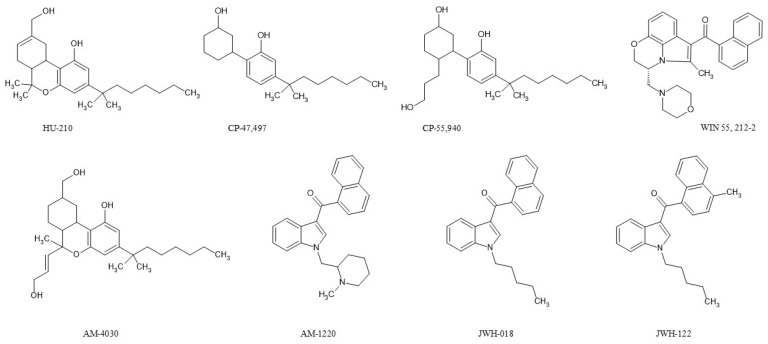
Examples of the different structural classes of synthetic cannabinoids.

Nomenclature of SCs is challenging because of their structural complexity. There are several nomenclature systems in use. Systematic chemical name describes their exact molecular structure, but it is complex and not suitable for routine communication or clinical use. The most common ones are serial names. The abbreviations can be derived from the initials of the scientists responsible for their first synthesis (e.g., ‘JWH’ series—John W. Huffman, ‘AM’ series—Alexandros Makriyannis) (Figure 1). They may also reflect the institution or pharmaceutical company where the compounds were originally developed (e.g., ‘HU’ series—Hebrew University of Jerusalem, ‘CP’ series—Carl Pfizer, ‘WIN’ series—Sterling-Winthrop company) (Figure 1) [3,6]. In 2011, the (EUDA, known until 2024 as EMCDDA) systematized the nomenclature for SCs. This nomenclature describes each compound using a four-component model, which includes the ‘core’, ‘tail’, ‘linker’ and ‘linked group’. Each functional group has a unique code name derived from its long chemical name [3,6,7].

SCs mimic the effects of phytocannabinoids like Δ^9^-tetrahydrocannabinol (Δ^9^-THC), but they have a greater affinity to the receptors, resulting in more potent psychoactive effects than traditional substances. SCs may target different organs and affect the cardiovascular, digestive, neurological, pulmonary, and hepatic systems. The toxicity and high abuse potential of SCs could pose serious health risks to their users, so it is necessary to monitor trends in SCs usage [2,8]. Due to several limitations, such as difficulties obtaining real figures of the prevalence of drug abuse, an estimation of illicit drug usage is a significant challenge for drug surveillance systems and researchers.

The challenges posed by the SCs require innovative monitoring strategies like the analysis of untreated wastewater, known as wastewater-based epidemiology (WBE) [9,10]. Measuring human excretion products like SCs and their metabolites can provide objective information about community consumption for a defined catchment area. It enables the detection of temporal fluctuations almost immediately and does not require direct contact with individuals or collecting personal biological samples, thus preserving privacy [9,10,11]. WBE constitutes a helpful source of information for assessing drug consumption, complementary to the conventional estimation methods on drug use, and has become appreciated by international agencies like EUDA. Till now, WBE studies have included the classic illicit drugs, such as amphetamine-type stimulants, cocaine, opioids and THC [9,12]. Recently, NPS such as mephedrone, ketamine, and synthetic cathinones have been added to the range of compounds being determined in wastewater [13,14]; however, SCs are still not included.

This review focuses on the estimation of SCs use through wastewater analysis. We discussed and compared analytical methodologies applied for the detection and quantification of SCs in raw wastewater samples and summarized the results obtained so far regarding the SC content in urban wastewater. We paid special attention to challenges related to different experimental stages of WBE analysis that hinder the accurate measurement of SCs and their metabolites. We also described future perspectives in this emerging research field.

## 2. Methodology of the Literature Search

**Figure 2:**
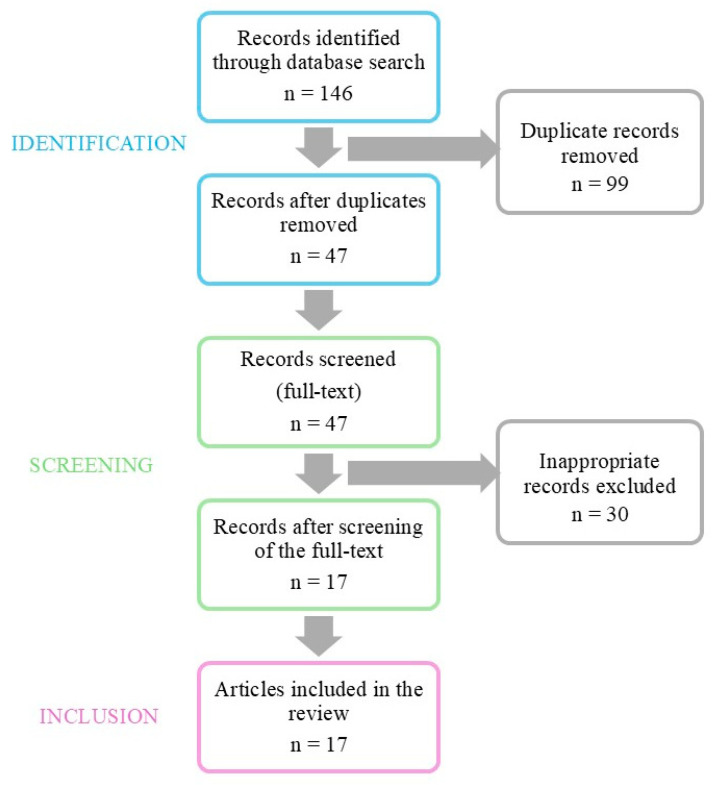
Chart of the literature search and selection process.

We conducted a comprehensive literature review in December 2025 to identify publications related to WBE studies on the detection and quantification of SCs. We limited our search to articles published between 2013 and 2025, because the first study in this field was published in 2013. Using keywords ‘Synthetic cannabinoids and wastewater’ or ‘Synthetic cannabinoids and sewage’, we searched Web of Science, Scopus, and PubMed databases. The search strategy yielded a total of 146 records. After removing 99 duplicates, we screened 47 articles. The selection was performed on the full text of the selected papers. After that, we excluded 30 studies that focused on the detection of other illicit drugs than SCs in wastewater samples or focused only on the detection of natural cannabinoids like Δ^9^-THC and its metabolites. After careful consideration, a total of 17 articles were included in this review. Figure 2 illustrates the screening and selection process for this systematic review.

## 3. Analytical Methodologies for the Analysis of SCs in Wastewater

A total of 16 analytical methodologies for the detection and quantification of illicit drug biomarkers in wastewater, including SCs, were reviewed across research published between 2013 and 2025 (Table 1 and Table S1).

Sampling strategies involved mainly 24 h composite, volume- or time-proportional collection of influent wastewater. The samples were typically stored at −20 °C or below to ensure analyte stability (Table 1 and Table S1).

Solid-phase extraction (SPE) using mixed-mode sorbents such as Oasis MCX or HLB (hydrophilic-lipophilic balance) cartridges represented the most common sample preparation approach (Figure S1) [15,16,17,18,19,20]. The choice of sorbent is determined by the dominant intermolecular interactions between the analyte and the stationary phase. MCX and HLB cartridges can extract a diverse range of analytes with varying properties. MCX provides high selectivity and recovery for extracting basic compounds. They are most widely employed due to their ability to retain protonated analytes. HLB cartridges can retain polar and non-polar compounds due to a combination of hydrophobic interactions, π-π interactions, and hydrogen bonding, which makes them highly adaptable for use over a broad pH range [21]. Despite the predominance of SPE in the reviewed WBE studies, the application of liquid–liquid extraction (LLE) was also reported in recent developments [18,22]. Magnetic solid-phase extraction (MSPE) and supramolecular solvent (SUPRAS) extraction were also described as a new approach to wastewater sample preparation for SC analysis [23,24].

The most often employed analytical technique was liquid chromatography tandem mass spectrometry (LC-MS/MS) with a triple quadrupole analyzer under positive electrospray ionization (ESI+) conditions [10,17,19,20,23,24,25,26]. This ‘gold standard’ is used in WBE due to its high sensitivity, selectivity, and suitability for targeted multi-residue determination in complex matrices. High-resolution mass spectrometry (LC-HRMS/MS) with an Orbitrap analyzer was occasionally used and primarily for confirmatory analysis or structural elucidation [16,27]. This system was used mainly in exploratory studies or in investigations focused on newly identified psychoactive substances, including SCs.

The most frequently applied chromatographic stationary phases were biphenyl, C_18_, and PFP (pentafluorophenyl) columns, with formic acid or ammonium formate modifiers to optimize separation and ionization efficiency [10,16,17,18,19,23,24,25,26,28,29]. The selection of sorbent and solvents depends on the predominant types of intermolecular forces involved in the system, guiding how well the analytes interact with the stationary and mobile phases during chromatographic separation. The SCs are a widely diversified group with different chemical properties, which hinder the selection of the appropriate chromatographic conditions. Generally, they are lipophilic and do not have strong acidic or basic properties. Due to the moderate to high lipophilicity of SCs, C_18_ phase columns are preferred, which provide strong hydrophobic interactions, giving good separation and reproducible retention times. However, for structurally similar analogues (e.g., positional isomers), C_18_ columns may not offer sufficient resolution. In such cases, PFP columns are used, which offer enhanced π-π interactions. That makes PFP columns attractive for aromatic compounds, improving the separation of positional isomers and structurally related fluorinated analogues [30]. Biphenyl columns combine the advantages of the classic C_18_ and PFP phases—they offer strong π–π interactions and moderate hydrophobicity. These phases provide improved selectivity toward polar metabolites, which exhibit weaker retention on purely alkyl-based stationary phases [31].

The vast majority of the studies cited described the validation procedure (Table 1 and Table S1). Reported validation parameters, such as linearity, limits of detection (LOD/ILOD), limits of quantification (LOQ/ILOQ), accuracy, precision, recovery, and matrix effects, showed that the methodologies were suitable for the intended purposes. However, the authors used different procedures during validation because no specific guidelines for WBE methodologies for the determination of illicit drugs have been developed yet [32]. The key validation parameters are LOD and LOQ values reflecting the sensitivity of the method. Only sufficiently low LOD and LOQ values allow the determination of SCs present in trace and ultra-trace amounts in wastewater. For instance, in the study by O’Rourke and Subedi [17], the LC-MS/MS method was validated for the simultaneous determination of 40 new psychoactive substances, including 11 SCs, in wastewater from four U.S. communities, achieving method detection limits (MDLs) ranging from 0.5 ng/L (for MMB-CHMICA) to 2.9 ng/L (MMB-FUBINACA).LOD for SCs spanned from 0.1 ng/L (5-fluoro EDMB-PINACA) to 6.3 ng/L (MAB-CHMINACA), while LOQ ranged from 0.4 (5-fluoro EDMB-PINACA) to 21.0 ng/L (MAB-CHMINACA) [17]. Similarly, in the study by Bade et al. [25], a direct injection LC-MS/MS method was optimized for quantifying 73 new psychoactive substances, including SCs, in Australian wastewater without prior extraction, achieving LOD as low as 0.2–5.5 ng/L and LOQ from 0.5–18.2 ng/L for SC analytes in influent samples. In the study by Borova et al. [28], a multi-residue LC-MS/MS method was developed for the determination of 10 new psychoactive substances, including SCs (i.e., JWH-group), in wastewater samples, achieving LOD in the range of 0.3–1.4 ng/L and LOQ from 0.9–4.4 ng/L depending on the analyte and matrix. Such performance underscores the method’s suitability for WBE, allowing reliable quantification of ultra-trace SCs in complex matrices without excessive false positives.

**Table 1:** Summary of the 16 methodologies of sampling, sample preparation and instrumental analysis aimed at the detection and quantification of synthetic cannabinoids and their metabolites in wastewater.

Sampling and Storage	Sample Preparation	Instrumentation	Validation	Reference
Raw wastewater sample 24 h mixed sampling	SPE Oasis MCX	UHPLC-MS/MS **Analyzer:** Triple quadrupole **Ionization:** ESI+ **Separation:** C_8_ column	Linearity, LOD, LOQ, accuracy, precision, recovery	[15]
Influent and effluent wastewater sample	SPE Spherical pure mixed polymer sorbent	LC-MS/MS **Analyzer:** Triple quadrupole **Ionization:** ESI+ and ESI− **Separation:** PFP column	Linearity, ILOD, ILOQ	[28]
Influent wastewater sample 24 h composite sampling	SPE Oasis MCX (150 mg)	LC-HRMS/MS **Analyzer:** ion trap-Orbitrap **Ionization:** DESI+ **Separation:** C_18_ column	Linearity, ILOD	[16]
Raw wastewater sample 24 h composite sampling	LLE **Solvent:** 10 mL HX: EtAc (1:1)	UHPSFC-MS/MS **Analyzer:** Triple quadrupole **Ionization:** ESI+ **Separation:** Torus 2-PIC Column	Linearity, MQL, IQL, precision, repeatability, trueness, matrix effects	[22]
Influent wastewater sample	SPE Mixed mode: C_8_+benzenesulfonic acid	LC-MS/MS **Analyzer:** Quadrupole time-of-flight **Ionization:** ESI+ **Separation:** PEP column	-	[33]
Raw wastewater sample 24 h composite sampling	SPE Oasis MCX (6cc, 150 mg)	LC-MS/MS **Analyzer:** Triple quadrupole ** Ionization:** ESI+ **Separation:** Biphenyl column	LOD, LOQ, precision, matrix effects, repeatability	[17]
Influent wastewater sample 24 h composite sampling	SPE Oasis HLB (6cc, 150 mg) LLE **Solvent:** 10 mL EtAc	LC-MS/MS **Analyzer:** Triple quadrupole ** Ionization:** ESI+ **Separation:** Biphenyl column	Linearity, accuracy, precision, matrix effects, selectivity, stability, recovery	[18]
Effluent wastewater sample containing 5% activated sludge	Filtration (0.2 μm, regenerated cellulose filters) Dilution (ultrapure water:MeOH 80:20)	LC-MS/MS **Analyzer:** Triple quadrupole ion trap ** Ionization:** ESI+ **Separation:** XSelect HSS T3 LC-HRMS **Analyzer:** Orbitrap **Ionization:** ESI+ **Separation:** XSelect HSS T3	Linearity, ILOD, ILOQ, matrix effects, recovery, precision	[27]
Influent wastewater sample 24 h composite sampling	SPE Cleanert PEP	LC-MS/MS **Analyzer:** Triple quadrupole **Ionization:** ESI+ **Separation:** C_18_ column	Linearity, recovery, sensitivity (LOD, LOQ), matrix effects, accuracy, precision	[10]
Influent wastewater sample 24 h composite sampling	SPE Oasis MCX (6cc, 60 mg)	LC-MS/MS **Analyzer:** Triple quadrupole ** Ionization:** ESI+ **Separation:** C_18_ column	Linearity, precision, sensitivity, matrix effects, recovery	[19]
Influent wastewater sample 24 h period sampling	Filtration: 10 mL of the sample (0.2 μm RC filter)	LC-MS/MS **Analyzer:** Triple quadrupole ** Ionization:** ESI+ **Separation:** Biphenyl column	Linearity, range, precision, LOD, LOQ, filtration losses, matrix effects	[25]
Influent wastewater sample 24 h composite sampling	Homogenisation Filtration (0.2 μm RC filter)	LC-MS/MS **Analyzer:** Triple quadrupole **Ionization:** ESI+ **Separation:** Biphenyl column	Selectivity, linearity, LOD, LOQ, accuracy, precision, matrix effects	[26]
Influent wastewater sample 24 h composite sampling	MSPE	LC-MS/MS **Analyzer:** Triple quadrupole **Ionization:** ESI+ **Separation:** C_18_ column	LOQ, linearity, accuracy, precision, matrix effects	[23]
Influent wastewater sample 24 h composite sampling	pH adjustment (pH 9) Filtration (PTFE filter)	UPLC-MS/MS **Analyzer:** Triple quadrupole **Ionization:** ESI+ **Separation:** Biphenyl column	LOD, recovery, selectivity, matrix effects	[29]
Influent wastewater sample	SUPRAS extraction	LC-MS/MS **Analyzer:** Triple quadrupole **Ionization:** ESI+ **Separation:** C_18_ column	Specificity, linearity, LOQ, accuracy, precision, recovery, matrix effects	[24]
Influent wastewater sample 24 h composite sampling	Filtration Acidification with HCl SPE Oasis MCX	LC-MS/MS **Analyzer:** Triple quadrupole **Ionization:** ESI+ **Separation:** PFP column	Linearity, precision, sensitivity, matrix effect, recovery	[20]

DESI+: desorption electrospray ionization in positive mode; ESI+: electrospray in positive mode; ESI−: electrospray in negative mode; EtAc: ethyl acetate; HX: hexane; ILOD: instrumental limit of detection; ILOQ: instrumental limit of quantification; IQL: instrumental quantification limits; LC-MS/MS: Liquid chromatography-tandem mass spectrometry; LLE: liquid–liquid extraction; LOD: limit of detection; LOQ: limit of quantification; MSPE: magnetic solid-phase extraction; MQL: method quantification limits; PFP: pentafluorophenyl; SPE: solid-phase extraction; SUPRAS: pentanol-type supramolecular solvents; UHPSFC-MS/MS: ultra-high performance supercritical fluid chromatography-tandem mass spectrometry.

## 4. Occurrence of SCs in Wastewater

Wastewater-based monitoring of SCs was reported across several continents, including Europe, Asia, Australia, and North America (Table 2), reflecting the global prevalence of these substances. Investigations conducted in 2014–2015 detected first-generation JWH-type cannabinoids (e.g., JWH-018, JWH-122, JWH-210) in Norway and Greece [15,28]. These findings provided some of the first evidence of SC usage in Europe. Subsequent studies performed between 2016 and 2020 identified a new group of fluorinated and indazole-based derivatives, including 5F-APINACA, AB-FUBINACA, MDMB-4en-PINACA, and UR-144 [10,18,33]. Thus, wastewater analysis reflected the dynamic evolution of the illicit SCs market.

Many recent studies from China conducted in 2020–2025 revealed a high diversity of detected SCs in wastewater samples collected from over 135 wastewater treatment plants in almost 40 cities (Table 2) [10,23,24,29]. These results highlight the variety and the constantly evolving SC market in East Asia. In contrast, investigations performed in Tunisia confirmed the presence of classical SC analogs in some regions, often with high prevalence [19,20]. This data suggests that the regional market of SCs may be less diversified than that of Europe or East Asia. This pattern reflects slower market evolution, possibly due to differences in distribution networks and the influence of socio-economic and regulatory factors.

It is also important to note that the majority of studies conducted to date have been limited to short-term monitoring periods. Only two investigations could be classified as long-term, as they encompassed wastewater samples collected over more than one month [10,29]. Consequently, the available datasets represent only a narrow temporal snapshot of SCs use within the studied populations. This restricted observational window limits the ability to capture seasonal fluctuations or episodic consumption patterns, underscoring the need for extended longitudinal monitoring to obtain a more comprehensive assessment of community-level exposure to SCs.

It should be noted that not all studies cited in Table 2 applied the population normalization for the determined concentration values of SCs. Only two studies performed by Reid et al. [15] and O’Rourke and Bikram Subedi [17] reported values expressed as mg/day/1000 inhabitants. Moreover, some studies were limited to only qualitative analysis and showed the results of the performed screening of SCs in wastewater samples (Table 2). This demonstrates that determination of SCs in wastewater is still in its infancy and further, intensified investigations are needed to obtain more detailed picture of SC use in communities.

Overall, the reviewed studies show that WBE represents a powerful and adaptable approach for monitoring emerging SCs, considering temporal and geographical trends in drug consumption. As analytical capabilities continue to evolve, particularly with the incorporation of LC-MS/MS, WBE is poised to become an indispensable tool in global drug monitoring and harm reduction strategies.

## 5. Challenges and Perspectives of Analysis of SCs in Wastewater

**Figure 3:**
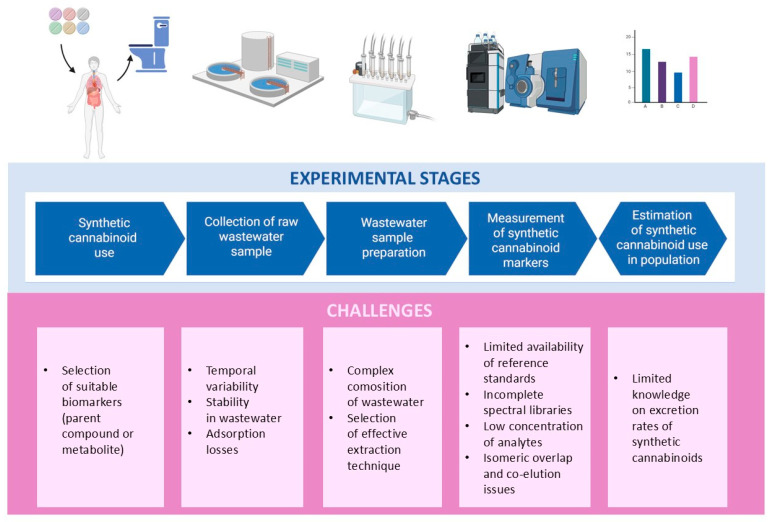
Challenges associated with the analysis of synthetic cannabinoids in wastewater at each experimental stage. Created in BioRender.com.

Analysis of SCs in wastewater faces challenges at each experimental stage (Figure 3). In addition, a great difficulty comes from the dynamic market of SCs and the large variety of their molecular structures. The chemical diversity of SCs demands the development of comprehensive multi-analyte methodologies covering a broad range of compounds and their metabolites. The rapid introduction of new compounds to the market requires updating the developed methodologies and optimizing them for newly released compounds [34].

Application of the WBE approach for SC analysis requires the selection of suitable biomarkers of consumption (parent compounds or metabolites), which are measured in raw wastewater. In evaluating the eligibility of a compound as a biomarker for monitoring purposes, its specificity, stability in urine and wastewater, as well as pharmacokinetics (i.e., metabolism and urinary excretion profile) should be considered [35]. The pharmacokinetic data are needed in WBE research for obtaining estimates of SC consumed by a population under investigation. In the absence of such data, only the concentration of a given drug or its metabolite in wastewater or their excreted mass loads (expressed in mg/day/1000 inhabitants) may be reported without estimating the amount taken by a given population. Information on metabolic pathways for many SCs is scarce, and a thorough investigation of their metabolism is needed [36]. SCs frequently exhibit limited stability (Table 3), potentially impacting their detection and quantification. Key factors influencing the possible compound biotransformation in sewage include microbial contamination, temperature, pH, and the amount of suspended particulate matter [37]. Methodologies used in WBE studies should rely on reliable target analytes, the selection of which should be preceded by stability tests involving the wastewater matrix.

The performed literature survey demonstrated that the stability of SCs and their metabolites in wastewater matrices varied considerably depending on chemical structure, storage temperature and duration, and sample treatment (Table 3). First generation of SCs, indole- and indazole-based SCs, including JWH-type compounds (e.g., JWH-018, JWH-210, JWH-398) and AM-series analogues (e.g., AM-2201, AM-2233), exhibited significant degradation under ambient and refrigerated conditions [16]. They degraded over time, even when frozen at −20 °C, with responses decreasing up to 67% after a week [16], likely due to adsorption to suspended solids or hydrolysis of ester or amide linkages inherent to many SC structures. Studies focusing on newer fluorinated indazole- and indole-derived SCs demonstrated improved short-term stability, particularly when samples were stored at 4 °C or below and treated with chemical preservatives (Table 3) [10,17,38]. The addition of sodium metabisulfite (Na_2_S_2_O_5_, 0.05% *w*/*v*) markedly extended analyte stability, preventing oxidative and hydrolytic degradation for up to 14 days [17]. Long-term storage (up to 120 days) at −20 °C or −80 °C maintained compound integrity for approximately one month, with significant signal decreases afterward [10]. Overall, the reviewed findings emphasize that storage temperature, pH, and the presence of antioxidants or preservatives are critical determinants of SC stability in wastewater. Immediate sample processing, or the addition of chemical stabilizers, should be considered essential for maintaining analyte integrity and ensuring data comparability across studies. The establishment of standardized stabilization protocols will be critical for improving the reproducibility and reliability of future WBE analysis targeting SCs. Despite performing the research summarized in Table 3, there are clear gaps in the existing knowledge and future studies should provide more answers about the long- and short-term stability of SCs.

One of the key uncertainties in monitoring drug use through sewage analysis is related to the sampling strategy and ensuring representativeness of the collected samples [39]. In the collection of raw wastewater samples, one should consider the temporal variability of SC levels in sewage. Fluctuations in influent wastewater composition over 24 h periods may affect representativeness. Therefore, 24 h composite sampling seems to be the best strategy in monitoring the consumption of illicit drugs. This strategy was applied in most of the reviewed studies (Table 1 and Table S1). Future studies should address the impact of different sampling strategies on the qualitative and quantitative data related to SC occurrence in wastewater. Fluctuations in weekly patterns of illicit drug loads were reported in several WBE studies, especially for ecstasy (3,4-methylenedioxymethamphetamine) and cocaine, reflecting typical recreational use patterns of those drugs [9,40,41]. Future WBE studies are needed to answer the question of weekly trends in SC use in the community. Another valuable sampling strategy is a collection of raw sewage during mass events, such as music festivals [42,43]. Analysis of sewage samples during carnival holiday in the Brazilian capital showed that consumption remained relatively constant, indicating that cannabis overall consumption is less affected by occasional abuse [44]. The summary of results presented in Table 2 demonstrates that studies of SC content in wastewater show only a snapshot of SC use in the community. The long-term monitoring of SC levels in wastewater should be performed to depict a broader perspective on SC consumption and investigate temporal trends in SC use by the community.

The major analytical challenge associated with the analysis of SCs in wastewater is related to their low concentrations. The consumption of SCs is lower in comparison with classic illicit drugs, and the choice is wide due to the numerous chemical modifications resulting in the formation of dozens of new compounds. Moreover, SCs and other new psychoactive substances are more potent than established drugs of abuse, which results in smaller doses being taken [45]. Thus, being excreted by consumers and heavily diluted in wastewater, SCs and their metabolites occur in wastewater at trace and ultratrace levels (Table 2). Therefore, WBE requires the application of highly sensitive analytical methods such as LC-MS for their analysis (Table 1 and Table S1). Future studies should address the impact of different sampling strategies on the qualitative and quantitative data related to SC occurrence in wastewater. The target quantitative methods employing a triple quadrupole analyzer provide excellent sensitivity and selectivity, but they are focused on a limited list of compounds for which reference standards are required [46]. Methods employing HRMS with Orbitrap or TOF analyzer offer many possibilities, from compound screening, the identification of new metabolites, to even quantification of compounds, but with a narrow analytical range and lower sensitivity [34,46]. Challenges in the measurement and reliable differentiation of structurally related SC biomarkers constitute a major analytical problem. Many analogues exhibit overlapping precursor ions and nearly indistinguishable fragmentation pathways, necessitating the use of advanced chromatographic and mass-spectrometric techniques. Recent studies have demonstrated that resolving positional isomers often requires detailed analysis of fragmentation pathways using high-resolution and multi-stage MS approaches. Misinterpretation can result in false positives or negatives [47,48,49].

The detectability and quantification of SCs in wastewater can be improved by using effective sample preparation techniques to isolate and concentrate the analytes. The predominant sample preparation technique employed in WBE studies is SPE [32]. However, developing a multi-analyte method suitable for a wide range of compounds, including classic illicit drugs, SCs, and other NPS, is challenging due to their different physicochemical properties. Due to a lower polarity and lipophilicity, and acidic character of their metabolites, cannabinoids are not always determined using the same method as the other conventional illicit drugs [11,32,50]. Recently, LLE was presented as a cost-effective alternative to SPE of three cannabis biomarkers (THC, THC-OH, and THC-COOH) in raw wastewater [51]. A particularly critical factor in the sample processing stage is pH, as it influences analyte ionization state and binding affinity for a SPE sorbent (or partitioning in LLE phases). Moreover, changes in pH during extraction can alter analyte stability [38]. Research demonstrated that acidic conditions are unsuitable for accurate quantification of cannabinoids [18]. Acidification of the wastewater samples increases the stability of the majority of illicit drugs and their metabolites; however, it favors biotransformation of THC-COOH, the main urinary biomarker of cannabinoid consumption [46]. Another critical step in sample processing is filtration, which may cause possible analyte loss because of interactions with filter materials [50]. Research performed by Pandopulos et al. [18] showed that sample filtration using a glass microfiber filter lowered the recovery of most cannabinoids from wastewater. Therefore, SCs require an individualized approach and carefully selected conditions during the sample processing stage.

The WBE approach requires identification and quantification of the extremely low levels of SCs and their metabolites in an exceptionally complex matrix, namely, raw sewage. Untreated wastewater contains many interfering substances responsible for matrix effects, a common challenge in LC-MS. Moreover, the composition of wastewater can vary depending on the location or season [52,53]. Such complex environmental matrix composition poses a challenge in the development of selective and accurate methodologies of SC analysis. One should remember that wastewater matrix components may result not only in analyte signal suppression but also signal enhancement, which was reported during validation of WBE methodologies [19,25,26]. To mitigate matrix effects and improve the accuracy of the assay, different strategies can be applied, including selection of an effective extraction or another cleanup technique, dilution of the final extracts, improving chromatographic conditions to avoid co-elution of analytes with matrix components, and employing a stable isotope-labelled internal standard for each analyte [54]. Recently, a UPLC–MS/MS method employing SUPRAS extraction was proposed for the determination of 13 SCs in wastewater and hair, which showed negligible matrix effects in wastewater samples [24]. Advanced functional materials such as molecularly imprinted polymers and metal–organic frameworks have been shown to enhance selective extraction and pre-concentration of trace contaminants in complex aqueous matrices, addressing challenges of low abundance and matrix interference [55]. Their further development and integration into WBE workflows are expected to play an increasingly important role in the reliable monitoring of ultra-trace level substances, including SCs.

Beyond conventional mass spectrometry-based approaches, biosensors and bioassays are emerging as powerful complementary tools for the detection of illicit drugs in wastewater, offering rapid response, high sensitivity, and the potential for activity-based screening [56]. In particular, recent advances in nanomaterial-assisted biosensing have demonstrated the feasibility of detecting trace- and ultra-trace-level drug residues directly in complex wastewater matrices. A biosensor-based platform combining selective enrichment with signal amplification enabled the simultaneous detection of structurally diverse SCs at ultra-low concentrations, addressing key analytical challenges associated with their low abundance, rapid degradation, and structural heterogeneity [23]. Comparable biosensor concepts have been successfully applied to other classes of illicit drugs in WBE [57,58,59]. These studies demonstrate that biosensors, particularly those exploiting molecular recognition elements such as aptamers or receptor-based interactions, hold substantial promise for future WBE.

Another key issue in the analysis of SCs is the low availability of reference standards. Thus, compound identification using MS and MS/MS reference spectra, as well as accurate quantification, are challenging tasks. A lack of reference standards for many SCs and their metabolites results in incomplete spectral libraries. Poor coverage of novel analogues hinders confident annotation. The standards are also needed for method optimization (to set the parameters of a quantitative method in MS instruments with a triple quadrupole analyzer) and for validation purposes. To deal with the limited availability of standards of SCs and their metabolites, screening methodologies employing high-resolution mass spectrometry (HRMS) can be harnessed [34].

WBE provides a powerful population-level monitoring tool for assessing drug use and environmental contaminants. However, the collection and analysis of wastewater data raise important ethical and privacy considerations that warrant careful discussion. Although WBE does not target individuals, aggregated data may still carry implications for community-level privacy, stigmatization, and public perception. To address these issues, ethical boundaries, data anonymization procedures, and safeguards for privacy protection should be clearly defined and implemented. Several recent studies have highlighted these concerns and proposed frameworks for ethical WBE practices [60,61,62,63]. For example, guidelines emphasize the importance of structured ethical review processes, transparency in data reporting, and careful communication of findings to avoid unintended social consequences. By incorporating these ethical considerations, WBE studies can maximize public health benefits while minimizing potential risks related to privacy and social harm.

To sum up, future studies should address the main challenges in the wastewater analysis of SCs, including further stability tests, investigation of SC metabolism with in vitro or in vivo experiments, and studies of the selection of the biomarkers of SC consumption. In the development of WBE methodologies, special attention should be paid to several potential sources of underestimation of drug use, such as instability of SCs and their metabolites, losses during sample collection and preparation, and matrix effects. One should also not forget about potential sources of overestimation of results, such as overlapping fragment ions, in-source transformations or interfering matrix compounds causing ion enhancement. Carefully optimized analytical methods should be validated in inter-laboratory tests to prove their reproducibility and facilitate comparison of results between studies.

## 6. Concluding Remarks

The conducted studies highlight the potential of wastewater analysis as a near-real-time, non-invasive surveillance tool for monitoring emerging SCs and assessing population-level drug exposure. The established analytical workflows employed in WBE studies should be optimized for the analysis of SCs. Modern analytical hyphenated systems such as LC-MS are essential for the sensitive and accurate quantification of SC biomarkers in wastewater and their sound identification. As studies on the identification and quantification of SCs in wastewater remain scarce, large monitoring campaigns and research performed in more locations are needed. Ultimately, linking WBE with forensic and public health data could create an early-warning system for novel psychoactive substances, connecting analytical chemistry with population-level monitoring.

## Figures and Tables

**Table 2:** Summary of the main findings of studies aimed at the detection and quantification of synthetic cannabinoids and their metabolites in wastewater.

Country	Number of Tested WTPs/Number of Examined Cities	Year of Sample Collection	Quantified Compounds (Concentration)	Detected Compounds and Prevalence/Detection Frequency (% of the Samples)	Reference
Norway	3/3 (Oslo, Hamar, Bergen)	13 to 15 of July 2012	JWH-018 *N*-5-hydroxypentyl (83.4/157/160 ng/L)	JWH-018 *N*-5-hydroxypentyl; JWH-122	[15]
Greece (Santorini Island)	5/5 (Kamari. Fira, Karterados, Emporio, Ia)	July 2013	JWH-210 (3.7/2.7/1.9/2.4/1.5 ng/L); JWH-122 (2.8/1.6/4.1/1.5 ng/L); CP47, 497 (79/130/305/60/223/74/176/78 ng/L)	JWH-210; JWH-122; CP47, 497	[28]
Australia	50/-	August 2016	-	5F-APINACA; 5F-APINACA monohydroxylated; AM-2201; JWH-018; JWH-073; UR-144; UR-144 *N* pentanoic acid	[33]
USA	4/1 (Illinois)	August 2019	MAB-CHMINACA (2.5/4.0/4.4/0.6 ng/L)	MAB-CHMINACA	[17]
Australia	15/-	August 2019	-	5-fluoro ADB (5F-MDMB-PINACA); 5-fluoro APINACA; 5-fluoro APINACA N-(4-hydroxypentyl) metabolite; 5-fluoro PB-22; 5-fluoro PB-22 (3-carboxyindole); AB CHIMINACA; AB-CHMINACA metabolite M1A; AB-FUBINACA; AB-FUBINACA metabolite 2A; AB-PINACA; AB-PINACA N-(4-hydroxypentyl) metabolite; AKB48 N-(5-hydroxypentyl) metabolite; AM-2201; AM-2201 (6-hydroxyindole) metabolite; APINACA (AKB48); JWH-018; JWH-018 (5-hydroxypentyl) metabolite; JWH-073; JWH-073 (4-hydroxybutyl) metabolite; MDMB-CHMICA; PB-22; PB-22 N-(4-hydroxypentyl) metabolite; UR-144; UR-144 N-(5-hydroxypentyl) metabolite; XLR-11; XLR-11 N-(4-hydroxypentyl) metabolite	[18]
China	135/31	July 2020–January 2021	ADB-BUTINACA (1.9 ng/L); 4-Fluoro MDMB-BUTICA butanoic acid metabolite (3.1 ng/L); 4-Fluoro MDMB-BUTINACA (0.1 ng/L); 5-Fluoro AMB metabolite 3 (0.4 ng/L); 5-Fluoro EMB-PICA (0.09 ng/L); 5-Fluoro MDMB-PICA (0.5 ng/L); 5-Fluoro MDMB-PICA metabolite 7 (29.1 ng/L); MDMB-4en-PINACA (0.2 ng/L); MDMB-4en-PINACA butanoic acid metabolite (72.1 ng/L)	ADB-BUTINACA (3.6%); 4-Fluoro MDMB-BUTICA butanoic acid metabolite (3.1%); 4-Fluoro MDMB-BUTINACA (2.1%); 5-Fluoro AMB metabolite 3 (0.2%); 5-Fluoro EMB-PICA (1.0%); 5-Fluoro MDMB-PICA (2.5%); 5-Fluoro MDMB-PICA metabolite 7 (0.2%); MDMB-4en-PINACA (4.0%); MDMB-4en-PINACA butanoic acid metabolite (13.4%)	[10]
Tunisia	3/3 (Choutran, Charguia. Rades Sud Meliane)	November 2019	-	JWH-250 (20%); CP 47, 497 (71%); HU-210 (9%)	[19]
China	-/1	-	JWH-307 (0.28 ng/L); CH-FUPIATA (0.679/0.834 ng/L)	JWH-307; CH-FUPIATA	[23]
China	-/1	July 2023–June 2024	-	MDMB-INACA (0.3%); FUBIMINA N-(5-hydroxypentanyl)M (0.1%); AB-FUBINACA M3 (0.5%); 5-fluoro AMB M7 (0.1%); 5F-AMB (0.1%); 5F-ADB M7 (0.2%)	[29]
China	5/5	-	-	5F-MPP-PICA (15.4%); AMB-4en-PICA (2.5%); 5F-MDA-19 (2.5%); 5F-MDMB-PICA (12.8%); ADB-CHMINACA (38.5%); 4F-MDMB-BUTINACA (7.7%); MDMB-4en-PINACA (41%); FUB-APINACA (5.1%)	[24]
Tunisia	5/1 (Sfax)	November 2021	-	JWH-398 (41.9%); JWH-250 (71%); JWH-018 (22.6%); HU-210 (12.9%); CP47, 497 (96.8%)	[20]

**Table 3:** Summary of studies on the stability of synthetic cannabinoids in wastewater.

Tested Compounds	Conditions	The Main Findings	Reference
JWH-007; JWH-016; JWH-019; JWH-081; JWH-098; JWH-122; JWH-147; JWH-203; JWH-210; JWH-251; JWH-302; JWH-307; JWH-398; AM-694; AM-2201; RCS-4; RCS-8; CB-13; AM-2233	Evaluated in different matrices, at different temperatures, and during different time frames: ➢ wastewater SPE extracts 4 °C for 24 h −20 °C for 1 week ➢ spiked ultrapure water 4 °C for 24 h −20 °C for 1 week ➢ 0.1 ng/μL standard in a solution of ultrapure water/MeOH 80:20 (*v*/*v*) 4 °C for 24 h	Concentrations of synthetic cannabinoid in wastewater extracts were fairly stable for 24 h at 4 °C (response decrease below 20%) but not after 1 week at −20 °C (response decrease up to 67%). Concentrations of synthetic cannabinoids in ultrapure water decreased up to 80% after 24 h at 4 °C and up to 70% after 1 week at −20 °C. In a solution of ultrapure water/MeOH, for most of the analites, the signal decrease varied less than 30%, except for JWH-147 (66%), JWH-210 (50%), JWH-398 (45%), AM-694 (53%), CB-13 (41%), and AM-2233 (53%).	[16]
5-fluoro ADB (5F-MDMB-PINACA); 5-fluoro APINACA; 5-fluoro APINACA N-(4-hydroxypentyl) metabolite; 5-fluoro PB-22; 5-fluoro PB-22 (3-carboxyindole); AB CHIMINACA; AB-CHMINACA metabolite M1A; AB-FUBINACA; AB-FUBINACA metabolite 2A; AB-PINACA; AB-PINACA N-(4-hydroxypentyl) metabolite; AKB48 N-(5-hydroxypentyl) metabolite; AM-2201; AM-2201 (6-hydroxyindole) metabolite; APINACA (AKB48); JWH-018; JWH-018 (5-hydroxypentyl) metabolite; JWH-073; JWH-073 (4-hydroxybutyl) metabolite; MDMB-CHMICA; PB-22; PB-22 N-(4-hydroxypentyl) metabolite; UR-144; UR-144 N-(5-hydroxypentyl) metabolite; XLR-11; XLR-11 N-(4-hydroxypentyl) metabolite	Collected over 14 days (left for 0, 1, 2, 3, 7, 14 days). Stored at: room temperature 4 °C Evaluated with: no treatment addition of Na_2_S_2_O_5_ (0.05% *w*/*v*) addition of HCl (pH 2)	At room temperature, the majority of cannabinoids were not stable in untreated wastewater for up to 14 days. Storing samples at 4 °C greatly improved analyte stability. The addition of a preservative extends analyte stability in wastewater. Storing samples at a lower temperature with a preservative improved the stability of all analytes for up to 14 days.	[28]
ADB-BUTINACA; ADB-BUTINACA N-(4-hydroxybutyl) metabolite; ADB-BUTINACA N- butanoic acid metabolite; 4-Fluoro MDMB-BUTICA; 4-Fluoro MDMB-BUTICA butanoic acid metabolite; 4-Fluoro MDMB-BUTICA N-(4-hydroxybutyl) metabolite; 4-Fluoro MDMB-BUTINACA; 5-Fluoro ADBICA; 5-Fluoro AMB metabolite 3; 5-Fluoro EMB-PICA; 5-Fluoro EMB-PICA N-(hydroxypentyl) metabolite; 5-Fluoro MDMB-PICA; 5-Fluoro MDMB-PICA metabolite 7; FUB-144; MDMB-4en-PINACA; MDMB-4en-PINACA butanoic acid metabolite	Collected over 120 days with short intervals (0, 2, 4, 6, 8, 12, 24, 48, 96 h) and longer intervals (7th, 14th, 30th, 60th, 120th day) in triplicate. Stored at: room temperature (20 °C) 4 °C −20 °C −80 °C	Good stability for tested compounds within 24 h, even at room temperature (exceptions: 5-Fluoro EMB-PICA and 5-Fluoro EMB-PICA N-(hydroxypentyl) metabolite—decrease in concentration by over 40% and 20% at room temperature after 24 h; good stability at 4 °C). The majority of substances were stable at −20 °C and −80 °C for 30 days. Significant decrease in concentration after long-term storage.	[10]
AB-PINACA; APINACA 4-hydroxypentyl; 5F-APINACA 5-hydroxypentyl	Conditions for 28-day experiment: ➢ room temperature (20 °C) ➢ refrigerator temperature (3–6 °C) ➢ addition of sodium metabisulfite ➢ pH 2	Within 7 days under each storage condition, the concentration of each synthetic cannabinoid decreased to approximately 6–7% of its original concentration for room temperature, pH 2, sodium metabisulfite, and 1% for refrigerator temperature.	[38]

## Data Availability

No new data were created or analyzed in this study. Data sharing is not applicable to this article.

## Supplementary Materials

The following supporting information can be downloaded at: https://www.mdpi.com/article/10.3390/molecules31020337/s1, Figure S1. Solid phase extraction scheme: (a) Conditioning, (b) Sample loading, (c) Washing, (d) Analyte elution. Table S1. Summary of the 16 methodologies of sampling, sample preparation and instrumental analysis aimed at the detection and quantification of synthetic cannabinoids and their metabolites in wastewater.
