# Supplementary material for: Data Hidden in Sewage: Advanced Methods for Identification and Quantification of Synthetic Cannabinoids in Urban Wastewater
Source: Molecules. 2026 Jan 19;31(2):337. doi: 10.3390/molecules31020337 (PMC12844115; doi:10.3390/molecules31020337)
Supplement: Supplementary file 1 [file molecules-31-00337-s001.zip › molecules-4066364-supplementary.pdf]

Data Hidden in Sewage: Advanced Methods for Identification and Quantification of Synthetic Cannabinoids in Urban Wastewater

Wiktoria Kurzeja [1], Mariola Kuczer [1], Jan Matysiak [2] and Agnieszka Klupczyńska-Gabryszak [2,*]

[1] Faculty of Chemistry, University of Wrocław, F. Joliot-Curie 14, 50-383 Wrocław, Poland; wiktoriakurzeja23@gmail.com (W.K.); mariola.kuczer@uwr.edu.pl (M.K.)

[2] Department of Inorganic and Analytical Chemistry, Faculty of Pharmacy, Poznan University of Medical Sciences, Rokietnicka 3, 60-780 Poznań, Poland; jmatysiak@ump.edu.pl

* Correspondence: aklupczynska@ump.edu.pl; Tel.: +0048-61-641-83-38

SUPPLEMENTARY MATERIALS

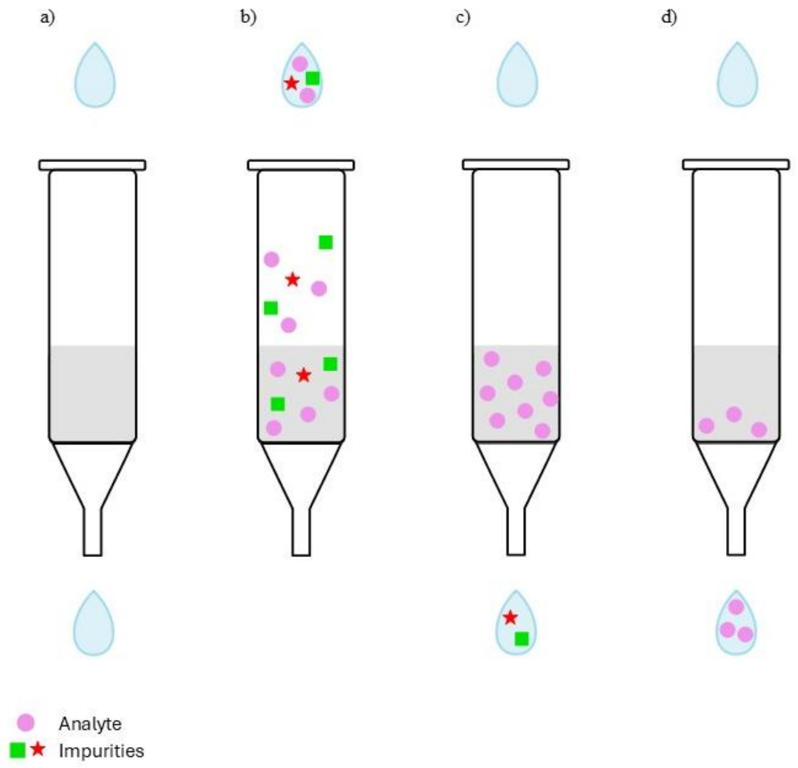

Figure S1. Solid phase extraction scheme: a) Conditioning, b) Sample loading, c) Washing, d) Analyte elution.

Table S1. Summary of the 16 methodologies of sampling, sample preparation and instrumental analysis aimed at the detection and quantification of synthetic cannabinoids and their metabolites in wastewater.

Sampling and storage	Sample preparation	Instrumentation	Validation	Reference
Raw wastewater sample<br>24 h mixed sampling<br>Volume-proportional sampling<br>Stored at -20°C	SPE<br>Oasis MCX<br>**C:** 12 mL MeOH, 12 mL acidified MilliQ water (0.1% AA at pH 3)<br>**SL:** 500 mL sample<br>**W:** 12 mL acidified water (0.1% AA at pH 3)<br>**E:** 12 mL of 2% NH_4OH in MeOH and 6 mL ACN<br>**R:** 2 mL of mixture ACN, MeOH, and MilliQ water	UHPLC-MS/MS<br>**Analyzer:** Triple quadrupole<br>**Ionization:** ESI+<br>**Separation:** C_8 column<br>**Injection volume**: 7 µL<br>**Solvent A:** 10mM ammonium acetate and 0.1% AA in water<br>**Solvent B:** 10mM ammonium acetate and 0.1% AA in MeOH	Linearity<br>LOD<br>LOQ<br>Accuracy<br>Precision<br>Recovery	[15]
Influent and effluent wastewater sample<br>Stored at 4°C	SPE<br>Spherical pure mixed polymer sorbent<br>**C:** 6 mL MeOH, 6 mL of acidified ultrapure water (HCl, pH 2.5)<br>**SL:** 50 mL sample<br>**W:** 3 mL ultrapure water (pH 2.5)<br>**E:** 2x2 mL MeOH and 4x2 mL MeOH:EtAc (50:50)<br>**R:** 500 µL MeOH:water (60:40)	LC-MS/MS<br>**Analyzer:** Triple quadrupole<br>**Separation:** PFP column<br>**Injection volume:** 10 µL<br><br>**Ionization:** ESI+<br>**Solvent A:** 0.2% v/v FA in MilliQ water<br>**Solvent B:** MeOH<br><br>**Ionization:** ESI-<br>**Solvent A:** MeOH<br>**Solvent B:** ACN	Linearity<br>ILOD<br>ILOQ	[28]
Influent wastewater sample<br>24 h composite sampling<br>Volume- or time-proportional sampling<br>Stored at -80°C	SPE<br>Oasis MCX (150 mg)<br>**SL:** 200 mL sample<br>**W:** 6 mL sodium acetate buffer, 2 mL of 0.1 M AA and 6 mL MeOH<br>**E:** 3 mL MeOH and 3 mL of 2% NH_4OH in MeOH<br>**R:** ultrapure water:MeOH (80:20)	LC-HRMS/MS<br>**Analyzer:** ion trap-Orbitrap<br>**Ionization:** DESI+<br>**Separation:** C_{18} column<br>**Injection volume:** 2 µL<br>**Solvent A:** 0.1% FA in ultrapure water<br>**Solvent B:** ACN	Linearity<br>ILOD	[16]
Raw wastewater sample	LLE<br>**Solvent:** 10 mL HX: EtAc (1:1)	UHPSFC-MS/MS<br>**Analyzer:** Triple quadrupole	Linearity<br>MQL	[22]

24 h composite sampling<br><br>Stored at -20°C		**Ionization:** ESI+<br>**Separation:** Torus 2-PIC Column<br>**Injection volume:** 2 µL<br>**Mobile phase:** CO_2<br>**Cosolvent:** 0.1% FA in MeOH:iPrOH (1:1 v/v)	IQL<br>Precision<br>Repeatability<br>Trueness<br>Matrix effects	
Influent wastewater sample<br><br>Stored at -20°C	SPE<br>Mixed mode: C_8+benzenesulfonic acid<br>**C:** 6 mL MeOH, 6 mL of 20 mM sodium acetate buffer (pH 5)<br>**SL**<br>**W:** 6 mL sodium acetate buffer, 2 mL of 0.1 M AA and 6 mL MeOH<br>**E:** 6 mL dichloromethane: isopropanol:ammonia (80:16:4)<br>**R:** 20 µL of 0.1% FA in MeOH and 180 µL of 0.1% FA in MilliQ water	LC-MS/MS<br>**Analyzer:** Quadrupole time-of-flight<br>**Ionization:** ESI+<br>**Separation:** PEP column<br>**Injection volume:** 10 µL<br>**Solvent A:** water with 5% MeOH and 0.1% FA in water<br>**Solvent B:** MeOH with 5% water and 0.1% FA	-	[33]
Raw wastewater sample<br><br>24 h composite sampling<br><br>Time-proportional sampling<br><br>Stored at -20°C	SPE<br>Oasis MCX (6cc, 150 mg)<br>**C:** 3 mL MeOH, 3 mL water (pH~2)<br>**SL:** 100 mL sample<br>**E:** 5 mL 5% NH_3 in MeOH	LC-MS/MS<br>**Analyzer:** Triple quadrupole<br>**Ionization:** ESI+<br>**Separation:** Biphenyl column<br>**Injection volume:** 1 µL<br>**Solvent A:** 0.1% FA<br>**Solvent B:** MeOH	LOD<br>LOQ<br>Precision<br>Matrix effects<br>Repeatability	[17]
Influent wastewater sample<br><br>24 h composite sampling<br><br>Stored at -20°C	SPE<br>Oasis HLB (6cc, 150 mg)<br>**C:** 6 mL MeOH, 6 mL ultrapure water<br>**SL:** 20 mL sample<br>**W:** 6 mL of 5% MeOH in water (v/v)<br>**E:** 2 x 4 mL dichloromethane:isopropanol:ammonia (80:16:4)<br>**R:** 100 µL MeOH and 100 µL of 0.1% FA in ultrapure water<br><br>LLE<br>**Solvent:** 10 mL EtAc<br>**R:** 100 µL MeOH and 100 µL 0.1% FA in ultrapure water	LC-MS/MS<br>**Analyzer:** Triple quadrupole<br>**Ionization:** ESI+<br>**Separation:** Biphenyl column<br>**Injection volume:** 5 µL<br>**Solvent A:** 0.1% FA in ultrapure water and MeOH (95:5)<br>**Solvent B:** 0.1% FA in MeOH with water (95:5)	Linearity<br>Accuracy<br>Precision<br>Matrix effects<br>Selectivity<br>Stability<br>Recovery	[18]
Effluent wastewater sample containing 5% activated sludge	Filtration (0.2 µm, regenerated cellulose filters)	LC-MS/MS<br>**Analyzer:** Triple quadrupole ion trap	Linearity<br>ILOD	[27]

Stored at -26°C	Dilution (ultrapure water:MeOH 80:20)	**Ionization:** ESI+<br>**Separation:** XSelect HSS T3<br>**Injection volume:** 20 µL<br>**Solvent A:** ultrapure water with 5 mmol AF, pH 3<br>**Solvent B:** MeOH with 5% ultrapure water and 5 mM AF, pH 3<br><br>LC-HRMS<br>**Analyzer:** Orbitrap<br>**Ionization:** ESI+<br>**Separation:** XSelect HSS T3<br>**Injection volume:** 20 µL<br>**Solvent A:** ultrapure water with 5 mmol AF, pH 3<br>**Solvent B:** MeOH with 5% ultrapure water and 5 mM AF, pH 3	ILOQ<br>Matrix effects<br>Recovery<br>Precision	
Influent wastewater sample<br><br>24 h composite sampling<br><br>Time-proportional sampling<br><br>Stored at -20°C	SPE<br>Cleanert PEP<br>**C:** 3 mL MeOH, 3 mL water<br>**SL:** 50 mL sample<br>**E:** 3 mL MeOH<br>**R:** 250 µL MeOH	LC-MS/MS<br>**Analyzer:** Triple quadrupole<br>**Ionization:** ESI+<br>**Separation:** C_{18} column<br>**Injection volume:** 2 µL<br>**Solvent A:** 30 mM AF, 0.1% FA and ultrapure water<br>**Solvent B:** MeOH	Linearity<br>Recovery<br>Sensitivity (LOD, LOQ)<br>Matrix effects<br>Accuracy<br>Precision	[10]
Influent wastewater sample<br><br>24 h composite sampling<br><br>Stored at -20°C	SPE<br>Oasis MCX (6cc, 60 mg)<br>**C:** MeOH, MilliQ water and MilliQ water acidified at pH 2<br>**SL:** 50 mL sample<br>**W:** 5 mL of 2% NH_3 in water<br>**E:** 8 mL of a 2% NH_3 in MeOH<br>**R:** 1 mL of mixture MeOH:MilliQ water (10:90, v/v).	LC-MS/MS<br>**Analyzer:** Triple quadrupole<br>**Ionization:** ESI+<br>**Separation:** C_{18} column<br>**Injection volume:** 5 µL<br>**Solvent A:** 10 mM/L AF and 0.1% FA in water<br>**Solvent B:** 10 mM/L AF and 0.1% FA in MeOH	Linearity<br>Precision<br>Sensitivity<br>Matrix effects<br>Recovery	[19]
Influent wastewater sample<br><br>24 h period sampling<br><br>Time- or flow-proportional sampling	Filtration: 10 mL of the sample (0.2 µm RC filter)<br><br>Transfer of 1 mL filtrate into an LC vial with 12 µL ISTD (12.5 µg/ L)	LC-MS/MS<br>**Analyzer:** Triple quadrupole<br>**Ionization:** ESI+<br>**Separation:** Biphenyl column<br>**Injection volume:** 10 µL<br>**Solvent A:** MilliQ water:MeOH with 0.1% FA (95:5 (v/v))<br>**Solvent B:** MeOH:MilliQ water with 0.1% FA (95:5 (v/v))	Linearity<br>Range<br>Precision<br>LOD<br>LOQ<br>Filtration losses<br>Matrix effects	[25]

Stored at -20°C				
Influent wastewater sample<br><br>24 h composite sampling<br><br>Stored at -20°C	Homogenisation<br><br>Filtration: 10 mL of the sample (0.2 μm RC filter)<br><br>Transfer of 1 mL filtrate into an LC vial with 12 μL ISTD (12.5 μg/ L)	LC-MS/MS<br>**Analyzer:** Triple quadrupole<br>**Ionization:** ESI+<br>**Separation:** Biphenyl column<br>**Injection volume:** 10 μL<br>**Solvent A:** 95% ultrapure water, 5% MeOH and 0.1% FA<br>**Solvent B:** 95% MeOH, 5% ultrapure water and 0.1% FA	Selectivity<br>Linearity<br>LOD<br>LOQ<br>Accuracy<br>Precision<br>Matrix effects	[26]
Influent wastewater sample<br><br>24 h composite sampling<br><br>Stored at -20°C	MSPE<br>20 mg magnetic graphene oxide + 1 mL ionic liquid solution (20 mg/mL)<br>Mechanical stirring (30 min)<br>Separation with a magnet<br>**E:** 5 mL ACN<br>**R**: 100 μL initial mobile phase	LC-MS/MS<br>**Analyzer:** Triple quadrupole<br>**Ionization:** ESI+<br>**Separation:** C_{18} column<br>**Injection volume:** 5 μL<br>**Solvent A:** 0.1% FA in water<br>**Solvent B:** ACN	LOQ<br>Linearity<br>Accuracy<br>Precision<br>Matrix effects	[23]
Influent wastewater sample<br><br>24 h composite sampling<br><br>Flow-proportional sampling<br><br>Stored at -20°C	pH adjustment using aqueous-ammonia (pH 9)<br><br>900 μL sample + 100 μL of IS mix<br>Vortexing (30 s)<br><br>Filtration: 400 μL of the supernatant (PTFE filter)	UPLC-MS/MS<br>**Analyzer:** Triple quadrupole<br>**Ionization:** ESI+<br>**Separation:** Biphenyl column<br>**Injection volume:** 20 μL<br>**Solvent A:** 5 mMl/L ammonium acetate and 0.01% FA in water<br>**Solvent B:** ACN	LOD<br>Recovery<br>Selectivity<br>Matrix effects	[29]
Influent wastewater sample<br><br>Stored at -20°C	Filtration ("GE Whatman" nonadhesive, glass-fiber filter paper (Grade GF/CTM)).<br><br>Transfer of 10 mL wastewater samples into 15 mL polypropylene centrifuge tubes with 20 μL IS solution.<br>Vortexing.<br><br>Introduction of 1 mL SUPRASs into the centrifuge tube<br><br>Agitation at 40°C for 5 min on a table thermostatic oscillator, followed by centrifugation.	LC-MS/MS<br>**Analyzer:** Triple quadrupole<br>**Ionization:** ESI+<br>**Separation:** C_{18} column<br>**Injection volume:** 10 μL<br>**Solvent A:** 0.1% FA<br>**Solvent B:** ACN	Specificity<br>Linearity<br>LOQ<br>Accuracy<br>Precision<br>Recovery<br>Matrix effects	[24]

	Transfer of 0.4 mL of extracted SUPRASs into a 1.5 mL centrifuge tube.<br><br>Evaporation to dryness and subsequent reconstitution with 100 μL of MeOH:water (2:1, *v/v*).			
Influent wastewater sample<br><br>24 h composite sampling<br><br>Stored at -20°C	Filtration<br><br>Acidification with HCl<br><br>SPE<br>Oasis MCX<br>**C:** MeOH, MilliQ water, acidified MilliQ water (pH 2)<br>**SL:** 50 mL sample<br>**W:** 5 mL 2% NH_3 in water<br>**E:** 8 mL solution of 2% NH_3 in MeOH<br>**R:** 1 mL solution of MeOH:MilliQ water (10:90, v/v)	LC-MS/MS<br>**Analyzer:** Triple quadrupole<br>**Ionization:** ESI+<br>**Separation:** PFP column<br>**Solvent A:** 0.1% FA in water<br>**Solvent B:** 0.1% FA in MeOH	Linearity<br>Precision<br>Sensitivity<br>Matrix effect<br>Recovery	[20]

AA: acetic acid; ACN: acetonitrile; AF: ammonium formate; C: conditioning; DESI+: desorption electrospray ionization in positive mode; E: elution; ESI+: electrospray in positive mode; ESI-: electrospray in negative mode; EtAc: ethyl acetate; FA: formic acid; HX: hexane; ILOD: instrumental limit of detection; ILOQ: instrumental limit of quantification; iPrOH: isopropanol; IQL: instrumental quantification limits; LC-MS/MS: Liquid chromatography-tandem mass spectrometry; LLE: liquid-liquid extraction; LOD: limit of detection; LOQ: limit of quantification; MSPE: magnetic solid-phase extraction; MQL: method quantification limits; PFP: pentafluorophenyl; R: reconstitution; SL: sample loading; SPE: solid-phase extraction; SUPRAS: pentanol-type supramolecular solvents; UHPSFC-MS/MS: ultra-high performance supercritical fluid chromatography-tandem mass spectrometry; W: washing;
